# Does Practice Enhance Adaptability? The Role of Personality Trait, Supervisor Behavior, and Career Development Training

**DOI:** 10.3389/fpsyg.2020.594791

**Published:** 2021-02-04

**Authors:** Mei Mei, Fu Yang, Mingfeng Tang

**Affiliations:** School of Business Administration, Southwestern University of Finance and Economics, Chengdu, China

**Keywords:** learning goal orientation, incompetence accusations, career training, deliberate practice, career adaptability, early career scientists

## Abstract

Drawing upon career construction theory, we examined the mediating effect of deliberate practice (DP) on career adaptability (CA) and the effects of learning goal orientation (LGO) and supervisor incompetence accusations (SIA) as well as career development training (CDT) on DP. Using data collected from 204 Chinese PhD students in three waves over a period of 2 months, we found that individuals who were inclined to learn new skills and obtain new knowledge were more likely to deliberately practice professional activities in their fields. When a PhD student’s professional competence was questioned by his or her supervisor, the student was more prone to negative emotions and would reduce his or her effort in the development of expertise. CDT – contrary to expectations – negatively predicted DP of professional activities. One possible reason is that the participants in this study have strong autonomy so that those who really struggling are participating in training and seeking help and those who with strong professional abilities are not accessing training programs. Moreover, results showed that DP of professional activities significantly promoted PhD students to adapt to their academic circumstances. Implications for career-related practice within the academic domain are provided.

## Introduction

Career adaptability (CA), a central construct of career construction theory (Savickas, 2002; Savickas et al., 2009), represents individuals’ psychosocial resources that help one to fit environmental change (Savickas, 1997). Indeed, CA has been shown empirically to be a significant and universally valid factor that enables individuals to facilitate psychological adjustment and achieve adaptation goals (e.g., Guan et al., 2013; Fiori et al., 2015; Hirschi et al., 2015; Ghosh et al., 2019; Urbanaviciute et al., 2019), thus emerging vocational research on career construction theory has started to explore the drivers of CA (Coetzee and Harry, 2013; Zacher, 2014; Shin and Lee, 2016; Autin et al., 2017; Obschonka et al., 2018; Pajic et al., 2018).

Young scholars are a group that typically faces high levels of job insecurity and stress (Kinman, 2001; Levecque et al., 2017). The increased workloads and continuous change in academic research make universities increasingly stressful environments, resulting in a number of reports of mental health concerns such as depression and anxiety among PhD students (Levecque et al., 2017). In order to successfully adapt to occupational stressors and strains, PhD students need to be equipped with CA that could help them cope with current and anticipated occupational challenges. CA is considered as an effective human capital that enables individuals to reduce work stress (Johnston et al., 2013) and to form successful adaptation (Negru-Subtirica and Pop, 2016). In this respect, we emphasize the importance of CA in the academic context and focus on exploring the antecedents and underlying mechanisms of the development of CA.

Career construction theory and the ancillary research suggest that individuals who are open to new experiences tend to flexibly respond and adjust to career environments (Savickas, 2013; Tolentino et al., 2014). In order to explore the role of individual characteristics in predicting one’s adaptability, we focus on learning goal orientation (LGO), a relatively stable personality trait that refers to the tendency to develop competence and master new ideas (Dweck, 1986; Tolentino et al., 2014). In terms of work context, the influence of supervisor is one of the most frequently examined work characteristics (Levecque et al., 2017; Lan and Chen, 2020). Accusations of incompetence from PhD supervisors are negative events in which supervisors call into question the research abilities of the students, which may affect students’ negative emotions. Therefore, we attend to the role of supervisor incompetence accusations (SIAs) in affecting PhD students’ self-directed learning process. Moreover, from an organizational perspective, we focus on a supportive organizational environment in which organizations provide career guidance to individuals. As CA is arguably a dynamic and changeable construct rather than a relatively stable personal trait (Savickas and Porfeli, 2012), scholars suggested that the impact of career intervention should be considered in developing adaptability resources (Verbruggen and Sels, 2008). In the current research, we seek to examine career development training (CDT) as a potential antecedent of CA so as to see whether career interventions are effective for PhD students.

For PhD students, the primary tasks for their roles as students and young scholars are their academic research. Undoubtedly, the mastery of academic skills will affect PhD students’ career outcomes. In this respect, activities in terms of professional competence improvement may be important mechanisms through which PhD students could gain adaptability resources. Deliberate practice (DP) refers to a series of self-directed and repetitive activities (e.g., professional reading, mental simulation, and feedback seeking) that are performed in one’s area of expertise (Ericsson et al., 1993; Dunn and Shriner, 1999; Unger et al., 2009). In the present study, we further examine a mediating role of DP in the academic field and argue that PhD students may develop their CA through deliberately performing these professional activities.

## Theoretical Background and Hypothesis Development

### Career Adaptability

Career adaptability is defined as the “readiness to cope with the predictable tasks of preparing for and participating in the work role and with the unpredictable adjustments prompted by changes in work and working conditions” (Savickas, 1997, p. 254). It is viewed as a multidimensional concept organized into four types of psychological resources: concern, control, curiosity, and confidence (Savickas and Porfeli, 2012). These four constructs reflect individuals’ adaptive coping capacities that individuals use to respond to environmental change, which is affected by both individuals and the environment (Savickas, 1997; Savickas et al., 2009; Savickas and Porfeli, 2012). To date, some important strides have been made in identifying the influence of individual and contextual factors on CA (e.g., Guo et al., 2014; Hou et al., 2014; Tian and Fan, 2014; Xu and Yu, 2019). However, empirical evidence on the role of contextual factors in CA research is still somewhat scarce (Autin et al., 2017; Pajic et al., 2018). Accordingly, the present study attempts to explore both individual and contextual predictors of CA in the academic context.

### Deliberate Practice

Deliberate practice refers to a series of self-directed, effortful, and repetitive activities that have been specially designed to achieve expertise (Ericsson et al., 1993). As an essential learning behavior to attain an expert level of performance, DP has been emphasized and explored in various domains such as music (Ericsson et al., 1993), chess (Charness et al., 2005), sports (Hodges et al., 2006), software design (Sonnentag et al., 2006), and entrepreneurship (Unger et al., 2009; Keith et al., 2016; Ranabahu and Barrett, 2019). However, when it comes to scientific research, the role of DP to improve the current level of expert performance is neglected.

Deliberate practice consists of a wide range of practice and learning activities that are performed regularly. These regular, continuous, proactive learning activities facilitate remarkable adaptation across different domains in a changing environment (Ericsson et al., 1993; Ericsson and Lehmann, 1996). In previous studies, scholars have identified different forms of DP activities, for example, mental simulation, seeking feedback, consulting experts, or colleagues, etc. (Sonnentag and Kleine, 2000; Unger et al., 2009). When a specific activity is considered as DP, it needs to meet at least the following conditions: (a) the activity is performed on a regular basis; (b) the activity is designed for performance improvement; and (c) the activity is undertaken with the motive to improve and goes beyond the task requirements (Sonnentag and Kleine, 2000; Ericsson, 2006; Unger et al., 2009). Based on the previous research, the present study aims to examine the role of DP in scientific research field.

### Learning Goal Orientation and Deliberate Practice

According to Savickas and Porfeli (2012), individual differences serve as important antecedents in the career construction process. In this respect, we first focus on LGO, a dispositional personality that implies great willingness and persistence to try hard in the acquisition of knowledge and skills (Dweck, 1986). LGO is strongly associated with learning activities (Baek-Kyoo et al., 2013). Due to the mastery-oriented response pattern inherent in LGO (Dweck, 1986); individuals who are learning-goal-oriented are likely to engage in adaptive behaviors to improve competencies (Tolentino et al., 2014). In this respect, learning-goal oriented individuals tend to initiate DP including a series of adaptive behaviors designed to improve a person’s expert performance (Ericsson et al., 1993). Moreover, to deliberately train oneself to reach beyond one’s current level of performance, repeated attempts are required (Ericsson et al., 2007). LGO implies a high level of persistence in effort under difficult conditions (Dweck, 1986; Button et al., 1996), enabling individuals to endure massive amounts of practice on the specific activities. Thus, we hypothesize the following:

Hypothesis 1: LGO is positively related to DP.

### Supervisor Incompetence Accusations and Deliberate Practice

Incompetence accusations are “negative events in which fellow team members call into question the capability or performance of focal team members” (Chattopadhyay et al., 2010, p. 812). Consistent with the important role of leadership in work settings, the style of supervision also holds great promise for application in the academic context. Negative supervisor behaviors or events are likely to trigger subordinates’ negative emotions, whereby subordinates who received negative information from supervisors tend to produce negative behaviors toward their job (Hon et al., 2013). Because professional expertise is important for individuals to achieve legitimization of their profession and to get higher status in society (Chattopadhyay et al., 2010), people strive for positive performance assessment and recognitions of their professional competence. Incompetence accusations from PhD supervisors could be viewed as fairly negative information on academic tasks. It signals that a supervisor is dissatisfied with a PhD student’s professional ability. When PhD students perceive high levels of incompetence accusations, negative emotions, and pressure from such excessive criticism may reduce their efforts in accomplishing routine tasks (Hon et al., 2013). Therefore, PhD students would be discouraged to invest efforts in active learning when they receive serious incompetence accusations. Taken together, it is hypothesized:

Hypothesis 2: SIA is negatively related to DP.

### Career Development Training and Deliberate Practice

In the present study, we focus on CDT, as a key predictor of DP, referring to the experience in participating in multiple career activities including career interventions, career courses, and career counseling. It needs to be clarified that career-related training and DP have different contents. The purpose of career training is usually to improve vocational related skills. For example, through job interview role play, individuals can improve their career problem-solving abilities (Koen et al., 2012). In contrast, DP activities are conducted to improve one’s professional abilities (Ericsson et al., 1993). We theorize that individuals with CDT are likely to be encouraged to invest effort in DP. For example, career interventions usually include methods and task assignments such as career exploration, networking events, and role-playing exercises (Brown et al., 2003; Akkermans et al., 2014). Under the guidance of such experiences, individuals have chances to put their efforts into competence development practice after the interventions. Additionally, career courses can produce positive career outputs such as cognitive development, positive career-related thoughts, and career decision-making skills (Hansen et al., 2016). With the increased career abilities, one can better perform DP activities in his/her profession. Moreover, career counseling is found to be effective to help clients to improve their career self-directedness (Verbruggen and Sels, 2008), propelling individuals to engage in proactive learning activities. Thus, we hypothesize:

Hypothesis 3: CDT is positively related to DP.

### The Mediating Role of Deliberate Practice

As mentioned before, CA is arguably a malleable construct (Savickas, 1997; Savickas and Porfeli, 2012), suggesting that it may be developed through a series of training activities. DP involves a series of activities designed to improve the current level of professional performance (Ericsson et al., 1993), helps one update existing knowledge and better adapt to changing environments (Unger et al., 2009). These continuous and proactive learning activities have been examined to facilitate remarkable adaptation across different domains (e.g., Ericsson et al., 1993; Dunn and Shriner, 1999). Accordingly, we propose that DP is practical in the acquisition of essential CA resources in the career development process. For example, when individuals initiate learning activities to train themselves by constant feedback seeking, consulting, and professional reading, they may get access to available information about their professional strengths and weaknesses. This may lead them to look ahead to their future and better prepare for possible career opportunities, resulting in improved CA. Taken together, we propose that:

Hypothesis 4: DP is positively related to CA.

Based on the above argument, we hypothesize a mediating role of DP in transmitting the effects of LGO, SIA, and CDT on CA.

Hypothesis 5a: DP mediates the relationship between LGO and CA.
Hypothesis 5b: DP mediates the relationship between SIA and CA.
Hypothesis 5c: DP mediates the relationship between CDT and CA.

### The Present Study

**Figure 1:**
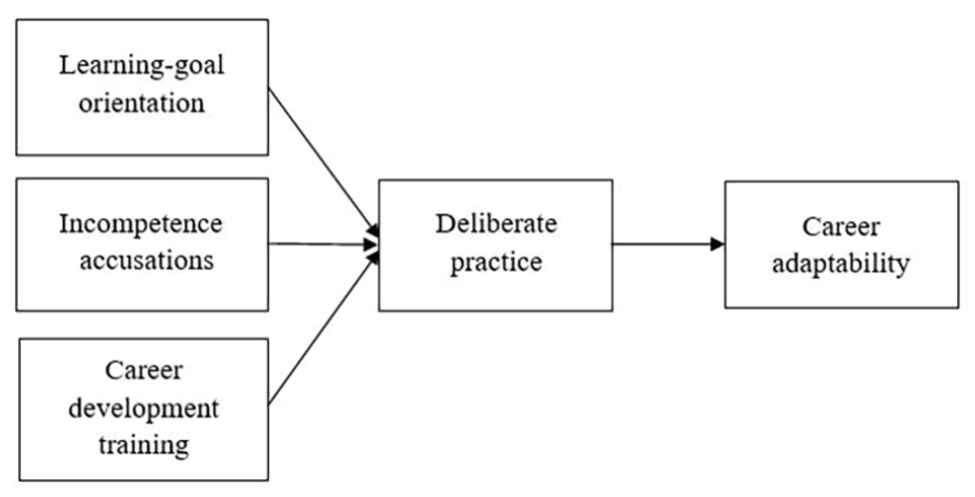
The theoretical model.

This study aims to make four primary contributions. First, drawing on career construction theory, our work extends the literature on the relationship between career adaptivity and CA. In career construction theory, adaptivity involves individual characteristics or the willingness to change (Savickas and Porfeli, 2012). We thus explore career adaptivity in terms of LGO among early career scientists, a group that especially needs frequent learning, and argue that LGO is an important antecedent of CA. Second, the investigation of incompetence accusations answered an expressed call in career construction research that the environment factors need to be taken into consideration in the analysis of CA (Savickas, 1997; Savickas et al., 2009; Savickas and Porfeli, 2012). Third, we aim to extend the literature on career-related training using a non-traditional sample of PhD students that are often overlooked in career development research. Finally, we offer a theoretical and empirical account of a self-learning factor (i.e., DP) as key mediating mechanisms, providing a better understanding of the role of self-directed learning in transmitting the effects of LGO, SIA, and CDT on CA. Figure 1 depicts the theoretical model of this study.

## Materials and Methods

### Participants and Procedures

Our participants were from two public universities in Southwest China. This research has been approved by the Academic Ethics Committee of Southwestern University of Finance and Economics. We collected research data in three separate waves with each wave about 1 month apart. In wave 1, participants were asked to complete measures of control variables (i.e., age, gender, grade, major, and graduation requirements) and independent variables (i.e., LGO, SIA, and CDT). A total of 277 participants completed the questionnaires in wave 1 (60% response rate). One month later, participants who completed wave 1 were asked to complete measures of the mediating variable (i.e., DP). In this round, we received a total of 239 completed questionnaires (86.3% response rate). Finally, 1 month after wave 2, participants who completed wave 1 and wave 2 were asked to evaluate their CA, resulting in 204 completed questionnaires (85.4% response rate).

We conducted the surveys in the two universities mainly using paper-based surveys, supplemented by some online responses. The paper and electronic questionnaires accounted for 72.6 and 27.4%, respectively. In order to collect continuous tracking data, we visited the PhD students’ offices to hand out the questionnaires. The online surveys were conducted through a widely used online survey platform in China. We sent out the questionnaire link through the social network of the research team to ensure reliable sources of data. The respondents of paper-based and online questionnaires were randomly selected in the first wave. To track respondents’ answers over time and match three rounds of data, we asked respondents to provide information on their initials and the last two of their student ID number. To encourage PhD students to participant in this investigation, students who completed each wave of the investigation were compensated with some cash or a small gift. Written informed consent was obtained from all participants in three waves in this study. All participants were assured that the participation was voluntary, and the survey was confidential.

Of the 204 participants who successfully completed the study, 50% were men and 78.4% were between 25 and 35 years old (*SD* = 0.48). Most students (81.9%) majored in Administrative and Economics. Majority (72.5%) were in the first and second PhD year. To avoid any potential effects of pressure, we controlled for whether the students have met requirements for graduation when performing the data analysis. Only 12.7% have met the basic requirements for graduation during the survey.

### Measures

The surveys were administered in Chinese. All the construct measures in the current study were originally developed in English. Following conventional back translation procedures (Brislin et al., 1973), the English measures were first translated into Chinese by a bilingual PhD student. Next, the Chinese items were translated back to English by a separate bilingual PhD student, who was blind to the research hypotheses and the original English items. Then, a bilingual professor of human resource management checked for inconsistencies between the original English language questionnaire and the back-translated one and found only minor discrepancies between these two versions of the measures. Finally, the translators and the professor discussed these discrepancies and made minor changes to the Chinese measures.

#### Learning Goal Orientation

We used the eight LGO items from the goal orientation scale developed by Button et al. (1996). All items used five-point Likert-type response scale with anchors from 1 = strongly disagree to 5 = strongly agree. Sample items are, “The opportunity to learn new things is important to me,” and “I do my best when I am working on a fairly difficult task.” The Cronbach’s alpha was 0.80.

#### Career Development Training

Following previous research on career training (Verbruggen and Sels, 2008), we used a dummy variable to measure career training participation in the current research. Previous scholars have used dummy variables to measure relevant training and education (e.g., Wilson et al., 2007; Entrialgo and Iglesias, 2016). Therefore, using a dummy variable to measure career training is acceptable in this study. Students who have participated in any career-related programming were coded as one, others were coded as zero.

#### Perceived Incompetence Accusations

We measured perceived incompetence accusations with four items adapted from a scale developed by Chattopadhyay et al. (2010). We amended the items that are originally used in the work context to be more specific to PhD students. Participants were asked to answer on a five-point Likert-type scale ranging from 1 = strongly disagree to 5 = strongly agree. Example items are, “My PhD supervisor conveyed messages that I needed to perform to a higher standard in academic research,” and “I received negative feedback about my research work from my PhD supervisor.” The Cronbach’s alpha was 0.70.

#### Deliberate Practice

We used eight DP activities identified in earlier research (Sonnentag and Kleine, 2000; Unger et al., 2009) to measure this variable. PhD students were asked to indicate retrospectively whether or not they have performed each given activity within the past month (0 = no, 1 = yes). The final score of DP for each respondent was measured using the total number of activities performed by them. Sample activities are, “Attending workshops/training” and “Professional reading.” The Cronbach’s alpha was 0.71.

#### Career Adaptability

The CAAS China form (Hou et al., 2012) was used to measure CA. Six items each for the subscales. Example items are, “Preparing for the future” (concern), “Sticking up for my beliefs” (control), “Investigating options before making a choice” (curiosity), and “learning new skills” (confidence). Participants were asked to indicate their career adaptabilities with response scale that range from 1 = not strong to 5 = strongest. The Cronbach’s alpha was 0.94.

#### Control Variables

Prior research has argued that socio-demographic factors such as age, gender, grade, and major have potential influence on students’ CA (e.g., Cai et al., 2015; Guan et al., 2017). For example, an empirical study has shown that CA would change over time (Koen et al., 2012). Thus, we controlled for PhD students’ age and year in program (YIP). Also, gender might play a role in the formation of CA because recent research suggests that gender differences exist on adaptability-related activities such as career exploration (Han and Rojewski, 2015), we therefore controlled for gender. The participants in this study were from a business university and a university of science and engineering. Because recent research suggests that the required abilities in the fields of Science, Technology, Engineering, and Mathematics are different from the business field (Wang and Degol, 2017), we also controlled for major using dummy variables (1 = business, 0 = non-business). In addition, previous research has suggested that pressure had influence on the adaptation process and the level of adaptability resources (Hou et al., 2014). Given that the pressure of doctoral students comes mainly from graduation requirements, we controlled for whether participants have met the basic academic requirements using a dummy variable (1 = have met the basic academic requirements, 0 = otherwise).

## Results

### Confirmatory Factor Analysis and Descriptive Statistics

Table 1 presents the descriptive statistics for study variables and controls. Before testing our hypotheses, we conducted confirmatory factor analysis to examine the distinctiveness of the four focal measures in the hypothesized model, namely, LGO, incompetence accusations, DP, and CA. As Pajic et al. (2018) elaborated CFI value greater than 0.85 is adequate. The results in Table 2 indicate that the hypothesized model is acceptable [*χ*^2^(129) = 245.00, *p* < 0.001, CFI = 0.90, TLI = 0.88, IFI = 0.90, and RMSEA = 0.07]. This measurement model fit the data better than constrained models (see Table 2). These results support discriminant validity of the hypothesized measurement model.

**Table 1:** Means, SDs, and correlations among variables.

S.no	Variable	*M*	*SD*	1	2	3	4	5	6	7	8	9
1.	Gender	0.50	0.50									
2.	Age	1.91	0.48	−0.14^*^								
3.	Major	0.82	0.38	0.00	0.18^**^							
4.	Year in program	1.96	1.00	−0.08	0.35^**^	0.12						
5.	Graduation requirements	0.13	0.33	0.09	−0.02	0.06	0.30^**^					
6.	Learning goal orientation	3.90	0.49	−0.05	−0.07	−0.07	−0.00	0.07				
7.	Incompetence accusations	2.80	0.73	−0.21^**^	0.09	0.11	0.05	−0.09	−0.04			
8.	Career development training	0.17	0.38	0.07	0.00	0.07	−0.06	0.10	0.04	0.04		
9.	Deliberate practice	4.18	2.19	0.03	−0.03	−0.02	−0.05	0.02	0.16^*^	−0.21^*^	−0.16^*^	
10.	Career adaptability	3.78	0.52	−0.13	0.09	0.02	0.16^*^	0.04	0.52^**^	−0.07	−0.04	0.21^**^

* *p* < 0.05

** *p* < 0.01.

*N* = 204.

**Table 2:** Confirmatory factor analysis results for the measurement models.

Model	*χ^2^*	*df*	*χ^2^/df*	CFI	TLI	IFI	RMSEA
Hypothesized four-factor model	245.00	129	1.90	0.90	0.88	0.90	0.07
Three-factor model: LGO + SIA, DB, CA	397.76	132	3.01	0.77	0.73	0.77	0.10
Two-factor model: LGO + SIA, DB + CA	430.61	134	3.21	0.74	0.70	0.74	0.10
One-factor model: LGO + SIA + DB + CA	589.97	135	4.37	0.60	0.55	0.61	0.13

*N* = 204. LGO, learning goal orientation; SIA, supervisor incompetence accusations; DP, deliberate practice; and CA, career adaptability.

### Hypothesis Testing

**Figure 2:**
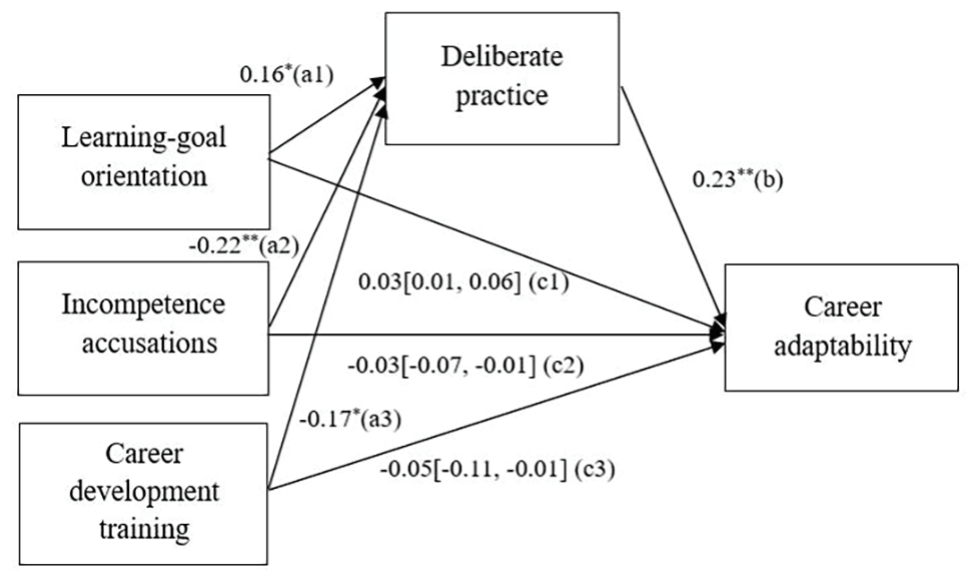
Summary of the regression results. ^∗^*p* < 0.05; ^∗∗^*p* < 0.01.

We examined the mediation effect with the procedure proposed by Preacher and Hayes (2008). First, we examined the mediating effect with LGO as predictor. The results of Model 2 in Table 3 showed that PhD students’ LGO significantly predicted their DP activities (*β* = 0.16, *p* < 0.05), supporting H1 (path a1 in Figure 2). The results of Model 6 suggested that DP was significantly related to CA (*β* = 0.23, *p* < 0.01), supporting H4 (path b in Figure 2). Then, we examined indirect effect by PROCESS analysis (Hayes, 2013) and the bootstrapping results (see Table 4) confirmed the indirect effect of DP on the link of LGO with CA [indirect effect = 0.03, boot se = 0.02, 95% CI = (0.01, 0.06); path c1 in Figure 2]. Overall, H5a was supported.

**Table 3:** Regression results of hierarchical regression analyses.

	Deliberate practice							Career adaptability				
Variable	M1	M2	M3	M4	M5	M6	M7	M8	M9	M10	M11	M12
Gender	0.02	0.03	−0.02	0.03	−0.12	−0.12	−0.08	−0.09	−0.14	−0.13	−0.12	−0.12
Age	−0.00	0.01	0.00	0.00	0.03	0.03	0.06	0.06	0.03	0.03	0.03	0.03
Major	−0.01	0.00	0.01	0.00	−0.00	0.00	0.03	0.03	0.01	0.01	0.00	0.00
YI P	−0.05	−0.05	−0.04	−0.07	0.14	0.16^*^	0.14^*^	0.15^*^	0.15	0.16^*^	0.14	0.16^*^
GR	0.03	0.02	0.01	0.05	0.01	−0.00	−0.04	−0.04	−0.01	−0.01	0.01	−0.00
LGO		0.16^*^					0.52^***^	0.50^***^				
SIA			−0.22^**^						−0.11	−0.06		
CDT				−0.17^*^							−0.02	0.02
DP						0.23^**^		0.15^*^		0.21^**^		0.23^**^
R2	0.00	0.03	0.05	0.03	0.04	0.09	0.31	0.33	0.05	0.10	0.04	0.09
ΔR2	0.00	0.03^*^	0.04^**^	0.03^*^	0.04	0.05^**^	0.27^***^	0.02^*^	0.01	0.04^**^	0.00	0.05^**^
F	0.15	0.98	1.66	1.05	1.69	3.30^**^	14.73^***^	13.82^***^	1.80	2.93^**^	1.42	2.82^**^

*N* = 204. YIP, year in program; GR, graduation requirement; LGO, learning goal orientation; SIA, supervisor incompetence accusations; CDT, career development training; and DP, deliberate practice.

* *p* < 0.05

** *p* < 0.01

*** *p* < 0.001.

**Table 4:** Results of PROCESS analyses.

Path	Effect	Boot se	95% CI (lower, upper)
LGO→DB→CA	0.03	0.02	(0.01, 0.06)
SIA→DB→CA	−0.03	0.02	(−0.07,−0.01)
CDT→DB→CA	−0.05	0.03	(−0.11,−0.01)

*N* = 204. LGO, learning goal orientation; SIA, supervisor incompetence accusations; CDT, career development training; DB, deliberate practice; and CA, career adaptability.

Second, we examined the mediating effect with SIA as predictor. Hypothesis 2 predicted that SIA is negatively related to DP. The results in Model 3 supported H2 (*β* = −0.22, *p* < 0.01; path a2 in Figure 2). PROCESS analysis (see Table 4) confirmed the indirect effect of DP on the relationship between SIA and CA [indirect effect = −0.03, boot se = 0.02, 95% CI = (−0.07, −0.01); path c2 in Figure 2]. Thus, H5b was supported.

Third, we examined the mediating effect with CDT as predictor. As shown in Model 4, CDT had a significant but negative relationship with DP (*β* = −0.17, *p* < 0.05; path a3 in Figure 2), contradicting H3. The results of PROCESS analysis indicated that DP had a mediating effect on the relationship between CDT and CA [indirect effect = −0.05, boot se = 0.03, 95% CI = (−0.11, −0.01); path c3 in Figure 2], thus H5c was supported. Table 4 presents the results of PROCESS analyses. The overall model and all the results are summarized in Figure 2.

## Discussion

### Antecedents of Deliberate Practice

Scholars have long emphasized the importance of DP in acquiring expert performance (Ericsson et al., 1993; Ericsson and Charness, 1994; Ericsson and Lehmann, 1996). To date, however, very few studies have clarified which factors may facilitate DP. A key contribution of the present study is to demonstrate that an individual difference factor (i.e., LGO) and a supervisor factor (i.e., SIA) as well as an organizational factor (i.e., CDT) serve as three important antecedents of DP and subsequent CA. Specifically, our results suggested that learning-goal-oriented PhD students tend to deliberately train themselves. The finding advances knowledge about the discussion of the relationship between inherent dispositions and DP in expertise research (Ericsson and Lehmann, 1996).

Second, our results clearly demonstrated that incompetence accusations from PhD supervisors reduced PhD students’ engagement in DP. Although researchers require constant criticism to help them improve the quality of their research, it seems that excessive or unfair accusations will reduce the research engagement and impair work performance of young scholars to a certain extent. This is similar to the negative role of abusive leadership in organizational research (e.g., Liu et al., 2012; Sungu et al., 2020).

Interestingly, we found that – contrary to our expectations – CDT was negatively related to DP and CA. A study of Spanish young drivers shows that the under-confident drivers are more interested in taking further training in safe driving than the over-confident ones (Molina et al., 2013). Accordingly, one possible reason why CDT was negatively related to DP and adaptability might be that the participants in this study have strong autonomy so that those who really struggling are participating in training and seeking help and those who with confidence are not involved in training programs. At first glance, one might assume that CDT would be positively associated with DP and CA. However, scholars have long argued that traditional career interventions may not be the best instrument to facilitate good adaptation in the long run due to the rapid change of practices in workplaces (Verbruggen and Sels, 2008). General career interventions such as repeated counseling sessions and assessments may be insufficient in the development of career resources (Koen et al., 2012). In addition, most of the existing CDT is designed for undergraduate students or employees, aiming at facilitating successful school-to-work or work-to-work transitions (e.g., Koen et al., 2012; Akkermans et al., 2014). The training program that works well for some career pathways (e.g., undergraduate level) may work less well for others such as PhD level scientific career pathways. Thus, we call for more studies on career training to specially design long-term interventions at a more granular level when promoting self-directed learning and relevant career competence.

### The Mediating Role of Deliberate Practice

The results showed that DP positively predicted CA. This indicates that PhD students may improve their CA through frequently performing professional-related activities, suggesting that DP serves as important learning behaviors in the adaptation process (Unger et al., 2009) within the academic domain. This finding extends previous knowledge about how CA could be enhanced through specific exercises by demonstrating DP as a key mediating mechanism. This is in line with the viewpoint that CA can be trained and thus it is indeed a learnable competence (Savickas et al., 2009; Koen et al., 2012). In addition, the findings show that DP is an important mediating mechanism linking three predictors (i.e., LGO, SIA, and CDT) with CA. Although existing research has examined extensive antecedents of CA from different perspectives, research on the underlying mechanisms in the form of adaptability competence is still rare. Our research extends the career construction literature by examining the mediating role of DP.

### Implications, Limitations, and Suggestions for Future Research

We deliberately used a sample of Chinese PhD students instead of the traditional sample of college and high school students when examining the underlying drivers of CA (e.g., Guo et al., 2014; Hou et al., 2014; Tian and Fan, 2014). PhD students are a special group that faces more and more competitive pressure and an increasing risk of mental health issues (Levecque et al., 2017). In our study, we adapted a DP scale specific to PhD students as many scholars assert the domain-specific nature of DP and found that DP is an effective way for doctoral students to attain adaptability resources. Future studies are encouraged to build on our findings to further test the effectiveness of DP in the career context.

This study also has several implications for practice. Our findings highlight the importance of DP in the academic context. Thus, the drivers of DP should be paid great attention in guiding PhD students toward increasing their involvement in self-improvement activities. First, the positive effect of LGO on DP calls attention on goal setting in the academic context. It seems valuable for PhD students and their supervisors to set learning goals toward the academic task and this may increase PhD students’ self-directed learning and lead to better adaptation. Second, our findings indicate that incompetence accusations from PhD supervisors reduce possible DP. It seems that PhD supervisors should encourage PhD students regularly to facilitate them to invest more effort in professional activities. Third, CDT failed to facilitate PhD students’ DP of professional activities in the current study. This is particularly noteworthy because it calls attention on rethinking and reform of traditional career training designed for general individuals. In this regard, we call for future research to examine the effectiveness of traditional interventions on PhD career development, Moreover, given that DP serves as an important driver in the form of CA, PhD supervisors could adopt the practice activities we identified in this study (e.g., regular workshop) to help PhD students initiate DP and improve professional competence.

Several limitations should be pointed out. First, in the current study, we tested the effect of career-development training on CA using a dummy variable. Although a large number of previous studies have adopted this method to measure training participation (e.g., Wilson et al., 2007; Entrialgo and Iglesias, 2016), as suggested by one of the reviewers, we still call for future research to use a scale variable to measure career training (e.g., number of training courses participated in, number of different types of nominal activities endorsed, and extent of engagement with career training). We also call for future study to apply a pre-test, a post-test, and a follow-up test to examine the effectiveness of training programs. Our second limitation is the relatively small sample size. Because our participants were only from two universities in the Southwestern part of China and the majority were from the business field, the generalizability of the findings to other regional and cultural groups and other fields outside of the business context is limited. Different cultural context provides different demands and opportunities to develop and express CA (Savickas and Porfeli, 2012). Thus, future research could make an attempt to apply the research findings to other cultural contexts and disciplines. Third, scholars have argued that self-report survey method may increase common method biases (Podsakoff et al., 2003). Although we collected data in three separate waves, there is still a possibility of common method biases due to the single source of data. Future research is encouraged to use multisource data. Finally, as qualitative research methods would allow participants’ lived experience to be explored through in-depth interviews, we suggest that future research conduct qualitative studies to shed additional light on how individuals feel and react when being accused of incompetence. Although our research contributes to the literature on antecedents of CA, it would also be an interesting avenue for further research to further discuss how adaptability affects the mental health of doctoral students.

## Conclusion

The present study extended the knowledge about the antecedents of DP and CA. We hope the theoretical implications gained through this study will encourage future research to investigate how to enhance individuals’ CA through targeted practice. Moreover, our focus on indirectly linking the three antecedents (learning-goal orientation, SIA, and CDT) to CA (through DP) also contributes to the research on the mediation role of DP in the expertise literature.

## Data Availability Statement

The raw data supporting the conclusions of this article will be made available by the authors, without undue reservation.

## Ethics Statement

The studies involving human participants were reviewed and approved by the Academic Ethics Committee of Southwestern University of Finance and Economics. The patients/participants provided their written informed consent to participate in this study. Written informed consent was obtained from the individual(s) for the publication of any potentially identifiable images or data included in this article.

## Author Contributions

MM and FY designed the study. MM analyzed the data and drafted the manuscript. FY revised the manuscript and contributed to the section “Discussion.” MT coordinated the data collection. All authors contributed to the article and approved the submitted version.

### Conflict of Interest

The authors declare that the research was conducted in the absence of any commercial or financial relationships that could be construed as a potential conflict of interest.
